# Hypoxic conditions confer chemoresistance to crizotinib but not to imatinib in chronic myeloid leukemia cells

**DOI:** 10.1016/j.htct.2025.106240

**Published:** 2026-01-10

**Authors:** Lena Avinery, Danielle Regev, Hazem Khamaisi, Jacob Gopas, Jamal Mahajna

**Affiliations:** a Department of Nutrition and Natural Products, Migal Galilee Research Institute, Kiryat Shmona, and department of Biotechnology, Tel-Hai College, Kiryat Shmona, Israel; b Shraga Segal Department of Microbiology, Immunology and Genetics, Ben Gurion University of the Negev, Beer Sheva, Israel

**Keywords:** Hypoxia, Chronic myeloid leukemia, Bcr/abl, Chemoresistance, Imatinib, Crizotinib, 2-methoxyestradiol

## Abstract

**Introduction:**

Chronic myeloid leukemia is an adult leukemia, constituting 15 % of all leukemia diagnoses. The fundamental driver of disease pathogenesis is the Bcr/Abl fusion protein, characterized by dysregulated tyrosine kinase activity. Abl kinase inhibitors have become the mainstay of treatment, however, patients often develop resistance due to genetic alterations, particularly affecting the Bcr/Abl oncoprotein. The tumor microenvironment is also associated with acquired resistance to Abl kinase inhibitors in chronic myeloid leukemia.

**Methods:**

The influence of hypoxic conditions on the development of chemoresistance to certain Abl kinase inhibitors was investigated in chronic myeloid leukemia.

**Results:**

This study showed that hypoxia increased resistance to crizotinib, while imatinib resistance was modest. Both drugs effectively inhibited Bcr/Abl activity. Interestingly, the JAK1/2 inhibitor ruxolitinib further enhanced chemoresistance to crizotinib under hypoxic conditions. Hypoxia-inducible factor 1α (HIF1α) overexpression in JAK2 knockdown experiments confirmed their cooperative role in mediating crizotinib resistance. In addition, 2-methoxyestradiol, a non-estrogenic estradiol metabolite, restored crizotinib sensitivity under hypoxia and the combination of 2-methoxyestradiol with a JAK2 inhibitor showed promising results in overcoming crizotinib resistance.

**Conclusion:**

In summary, this study shows the critical role of selective targeting of components of the HIF1α signaling pathway for the complete eradication of chronic myeloid leukemia cells.

## Introduction

Philadelphia chromosome-positive (pH^+^) leukemia is defined by the chromosomal translocation t(9;22), which leads to the formation of the *Breakpoint Cluster Region/Abelson proto-oncogene 1* fusion gene (*Bcr/Abl*). Persistent activation of *Bcr/Abl* leads to increased cell proliferation, decreased sensitivity to various apoptotic signals, and neoplastic transformation [1]. Abl kinase inhibitors (AKIs) are used to treat pH^+^ leukemia. Although these treatments are initially effective [2], their clinical efficacy declines with disease progression. Patients with chronic myeloid leukemia (CML) in blast crisis or acute lymphoblastic leukemia (pH^+^) rarely, if ever, benefit from AKI therapy [3]. Bcr/Abl-dependent or independent mechanisms may be responsible for resistance to therapy in pH^+^ leukemia. A significant contributing factor to drug resistance in pH^+^ leukemia is the bone marrow (BM) niche, which is a part of the tumor microenvironment (TME). The BM microenvironment is crucial for controlling stem cells and long-term hematopoiesis [4]. Nonetheless, soluble growth factors, interleukins, stromal cells, and extracellular components—all of which are found in the BM niche—may help reduce the susceptibility of cancer cells to treatment [5]. To overcome treatment failure in pH^+^ leukemia, new therapeutic approaches must be developed considering these drug resistance mechanisms mediated by the BM microenvironment.

The BM microenvironment can consist of areas of hypoxia (low oxygen) and acidity, which can interfere with the ability of chemotherapy to effectively treat tumor cells. Furthermore, hypoxia in the microenvironment has been associated with metastasis, malignant invasion, and a cellular transformation known as the epithelial-mesenchymal transition (EMT).

Both malignant and normal hematopoietic stem cells, including those from CML, may encounter a hypoxic niche in the BM microenvironment. High BM hypoxia levels have been linked to resistance to therapies in several malignancies [6], minimal residual disease persistence [7], and disease progression. A major factor in the course of the disease is the interaction between leukemic cells and their surroundings.

The interaction of leukemic cells with their environment plays a significant role in the progression of the disease. HIF-1α, an important regulator of the cellular response to low oxygen, is necessary for metabolic adaptability and proliferation under hypoxic conditions. HIF-1α stabilizes in hypoxic conditions and suppresses programmed cell death (apoptosis) by either directly or indirectly controlling the expression of several genes that mediate chemoresistance. The genes which have been shown to be regulated by HIF-1α include *Bak, Bax, Bcl-xL, Bcl-2, Bid, Mcl-1, NF-κB,* and *p53* [8]. Notably, whereas HIF-1α activation of anti-apoptotic target genes is well-established, the exact biological mechanisms underlying this phenomenon are still only partially known [9]. New data further emphasizes how the microenvironment mediates resistance to several Abl kinase inhibitors, including imatinib, nilotinib, and Dasatinib [10]. Furthermore, the microenvironment has been linked to the persistence of residual diseases of pH^+^ leukemia [11].

Even though imatinib actively inhibits Bcr/Abl kinase, pH^+^ leukemia cells under hypoxic conditions show partial suppression of apoptosis [12]. Given that BM microenvironment cells secrete soluble molecules including SDF-1, vascular endothelial growth factor (VEGF), and interleukin-6, which are all known to promote the survival of hematopoietic stem cells (HSCs), hypoxia may indirectly contribute to chemoresistance [13]. Chemotherapy resistance mediated by the BM microenvironment is still a major challenge in the treatment of leukemia.

The present study investigated how hypoxia affects the efficacy of the tyrosine kinase inhibitor crizotinib by exploring its off-target activities, rather than its established function as an anaplastic lymphoma kinase (ALK) inhibitor implicated in ALK-positive non-small cell lung cancer (NSCLC). This study aims to repurpose crizotinib for an additional therapeutic indication—specifically, to overcome chemoresistance in CML which is the focus of our research program.

## Materials and methods

### Chemicals and reagents

Sigma Aldrich Israel Ltd. (Rehovot, Israel) provided most of the chemicals, including cobalt chloride (CoCl_2_). The text includes specific sources for various compounds. 2-Methoxyestradiol (2ME2) was purchased from Cayman Chemical (Ann Arbor, MI, USA), Everolimus (Afinitor) and kinase inhibitors (Imatinib, Ruxolitinib, and Crizotinib) were obtained from Selleck Chemicals LLC (Houston, TX, USA).

### Cell lines

The American Type Culture Collection (ATCC, VA, USA) provided the human CML cell lines K562 and BV173. The cells were cultured in complete RPMI 1640 media supplemented with 0.1 mg/mL streptomycin, 100 units/mL penicillin, 1 % (w/v) l-glutamine, and 10 % (w/v) fetal bovine serum (Biological Industries, Israel). All cells were maintained in a humidified incubator with 5 % CO_2_ at 37 °C.

### HIF1α overexpression

Using a retrovirus harboring HA-HIF1α P402A/P564A (Addgene plasmid #1900 [14]), K562 cells were stably transfected to overexpress HIF1α, as previously reported [15]. In summary, puromycin selection was used to isolate stable transfected cells after K562 cells were infected with the retrovirus. Overexpression of HA-HIF1α was detected in the transfected cells using immunoblotting with anti-HIF1α antibodies.

### JAK2 silencing in K562 cells

Using a previously established CRISPR-Cas9 method, JAK2 expression was suppressed in K562 cells [15]. In summary, lenti-cas9blast (Addgene plasmid #52,962), gRNA JAK2 (Addgene plasmid #75,728), and lentiviral packaging plasmids (pcMV-dR8.2 dvpr and pcMV-VSV-G) (Addgene plasmids #8455 and #8454) [16] were co-transfected into HEK293T cells (1.5 × 10^5^ cells/mL) using Fugene 6 transfection reagent (Roche Applied Science, Penzberg, Germany) according with the manufacturer's instructions. The supernatant containing the lentiviral particles was collected 48 h post-transfection and utilized to infect K562 cells. Then, stably transduced clones with decreased JAK2 expression were isolated using blasticidin and puromycin selection. Western blotting was performed to confirm the downregulation of JAK2 protein levels.

### Cell viability assay

The trypan blue exclusion assay was used to assess cell viability. K562 cells were seeded at a density of 2 × 10^5^ cells per well in six-well plates. Cells were subjected to the specified treatments following a 24-hour incubation period. Dimethyl sulfoxide (DMSO) at a concentration of 1 % (w/v) was applied to the control samples. After exposure for 72 h, cells were collected, stained with a 0.4 % (w/v) trypan blue solution diluted 1:1 with culture media, and then manually counted using a hemocytometer according to the previous described protocol.

### Western blotting

Phosphatase inhibitors (AG Scientific, CA, USA) were used to produce cell lysates. After lysing the cell pellets in the buffer for half an hour on ice, the supernatants were separated by centrifugation. The DC™ Protein Assay (Bio Rad, USA) was used to determine the protein concentration in the supernatants (absorbance measured at 630 nm). Equal protein concentrations (50–60 µg each sample) were electrophoretically separated after being loaded onto 8–12 % polyacrylamide gels. After that, the proteins were transferred onto nitrocellulose membranes (Schleicher & Schuell BioScience GmbH, Germany). To decrease non-specific antibody binding, the membranes were then blocked using 5 % non-fat dried milk in Tris-buffered saline with Tween 20 (TBS-T).

To detect the presence of phospho-Abl (Tyr 245) and cleaved PARP (Poly(ADP-ribose) Polymerases - Cell Signaling Technology, MA, USA), α-tubulin (Santa Cruz Biotechnology, TX, USA), and phospho-ERK1/2 (Thr202/Tyr204) (Cell Signaling Technology, MA, USA), primary antibodies were employed. Every primary antibody incubation was carried out following the instructions recommended by the manufacturer.

Species-specific secondary antibodies combined with horseradish peroxidase (HRP), such as anti-mouse (NB7539, Novus Biologicals, CO, USA) and anti-rabbit (#7074, Cell Signaling Technology), were used to detect the bound primary antibodies. The SuperSignalTM West Pico PLUS chemiluminescent substrate (Thermo Fisher Scientific, MA, USA) was used following the manufacturer's instructions to accomplish chemiluminescent detection [17,18].

### Flow cytometry analysis

The expressions of CD45 and CD44 on the cell surface were evaluated by flow cytometry, as previously reported [15]. In short, K562 cells were resuspended at a density of 1 × 10^7^ cells/mL in phosphate-buffered saline (PBS) containing 0.5 % bovine serum albumin (BSA), together with K562/HIF1α and K562/Si JAK2 variations. The CD45 (KRO, #A96416, Beckman Coulter) and CD44 antibodies (FITC, #9011–0441, eBioscience) were either employed at the dilutions suggested by the manufacturer or titrated in advance to maximize staining. For forty minutes, the cell-antibody combination was incubated on ice. The labeled cells were analyzed using a Beckman Coulter Navios flow cytometer after washing with PBS mixed with 0.5 % BSA.

### RNA extraction and cDNA synthesis

Using Tri Reagent (Sigma), total RNA was isolated from cells following a previously described procedure [15]. The isolated RNA was then subjected to reverse transcription (RT), which produced single-stranded cDNA. In short, 15 µL of nuclease-free water (DEPC-treated) and 1 µL of oligo(dT)17 primer were added to 1 µg of RNA, and the mixture was pre-incubated for 10 min at 70 °C before being quickly cooled on ice. Second, 2 mL of 5X AMV RT reaction buffer, 2 µL of dNTP mix (25 mM), 28 units of RNasin ribonuclease inhibitor, 30 units of AMV reverse transcriptase, and nuclease-free water were added to the annealed primer-template mixture for a final volume of 10 µL. The RT reaction was incubated for 60 min at 42 °C.

### Polymerase chain reaction (PCR) amplification

A commercial PCR kit (Bioline, Taunton, MA, USA) was used to amplify the generated cDNA. Specific primers for each gene of interest are specified in Table 1. Thirty-five amplification cycles (each cycle: 94 °C denaturation for 30 s, 55–60 °C annealing for 30 s, and 72 °C extension for 2 min and 30 s) proceeded after the first denaturation step at 94 °C for two minutes. The cycling program concluded with a last extension step that lasted 15 min at 72 °C. Primers specific to the housekeeping gene β-actin were used as a control. The PCR products (5 µL) were separated by electrophoresis in a 1.5 % agarose gel.

**Table 1:** Oligonucleotide primers for gene amplification.

**Primers**	**Forward**	**Reverse**
hVEGF	5′-GTCGGGCCTCCGAAACCATG-3′	5′-CCTGGTGAGAGATCTGGTTC-3′
hBcl2	5′-CTGGAGAGTGCTGAAGATTGATG-3′	5′-CAATCACGCGGAACACTTGATTC-3′
hMcl1	5′-GATCAGTATATACACTTCAG-3′	5′-CAGGTGCAGCCTGTACTTGTC-3′
hβ-actin	5′-GCCCTGGACTTCGAGCAAGA-3′	5′-TGCCAGGGTACATGGTGGTG-3′

Sequences of the forward and reverse primers used for polymerase chain reaction along with their corresponding target genes.

### Quantitative real-time polymerase chain reaction (RT-PCR)

Quantitative RT-PCR amplification of mRNA transcripts was performed using the resultant cDNA. Each 15 µL reaction mixture included 1X SYBR GREEN reaction mix (Kappa Biosystems, Wilmington, MA, USA) and 0.2 pmol/µL of forward and reverse primers. A spectrofluorometric Rotor-Gene 6000 thermal cycler (Corbett Research, Mortlake, Australia) was used to perform the PCR. The Taq DNA polymerase was first activated by a 10-minute denaturation stage at 95 °C, which was followed by 55 cycles of denaturation at 95 °C for 15 s and a one-minute combined annealing/extension step at 55–60 °C. A range of cDNA template concentrations were evaluated to guarantee amplification within the linear range. As an internal control, the housekeeping gene β-actin was co-amplified in the same PCR reaction. The primer sequences employed in the semi-quantitative RT-PCR were the same.

### Colony formation assay

A clonogenic experiment was used to evaluate the capacity of cells to form colonies on a semi-solid medium, as previously reported [19]. In summary, K562 cells were diluted in complete RPMI 1640 media with 10 % fetal bovine serum (FBS) until they reached a density of 1 × 10^4^ cells/mL. The cell suspension was mixed with an equivalent amount of 0.6 % agar solution to reach a final agar concentration of 0.3 %. The cell-agar mixture was plated in 12-well plates over a layer of bottom agar that had already hardened and left to solidify. To maintain agar hydration after the top layer solidified, 1 mL of new medium containing the specified treatments was applied to each well. The cells were cultured for two weeks at 37 °C in a humidified environment with 5 % CO_2_ to enable colony formation. A colorimetric MTT (3-(4,5-dimethylthiazol-2-yl)-2,5-diphenyltetrazolium bromide) test was used to visualize and quantify colonies. The plates were incubated for 4 h with 5 mg/mL MTT solution, followed by the extraction of the formazan product using a solubilization buffer (20 % SDS, 50 % N,N-dimethylformamide, and 25 mM HCl). Using a plate reader, the optical density of the extracted dye was measured with a plate reader at 570 nm with a reference wavelength of 630 nm.

### Hypoxic conditions

A hypoxic atmosphere was created using a BioSpherix OxyCycler system (BioSpherix, Redfield, NY, USA). Cells were cultured in a custom-designed, computer-controlled incubator attached to the OxyCycler. A controlled environment of 37 °C, 5 % CO_2_, and 2 % O_2_ was maintained in the hypoxic chamber. The cells in the control group were cultured for the duration of the experiment in normoxic conditions (∼21 % O_2_ and 5 % CO_2_). Twelve hours was the incubation time for both normoxic and hypoxic experiments.

### Immunofluorescence staining and imaging

The cellular expression and location of phospho-c-Abl and HIF-1α proteins in K562 cells were determined by immunofluorescence. The immunofluorescence protocol involved coating a 96-well plate with poly-l-lysine (Sigma Aldrich Israel Ltd, Rehovot, Israel) four hours before the experiment completion and allowing it to dry for two hours. Following the seeding of 5 × 10^5^ cells in the coated wells, they were incubated for a further two hours under normoxic conditions and then 12 h of normoxic/hypoxic conditions as described above.

Following incubation, cells were washed twice with PBS and fixed with 4 % paraformaldehyde in PBS for 20 min at room temperature (RT). Following fixation, cells were washed twice with PBS and permeabilized using a solution containing 3 % FBS and 0.1 % Triton X-100 in PBS for 1 h at room temperature. Blocking was carried out for an extra one hour at room temperature using the same permeabilization solution. The primary antibody of interest (HIF1α or phospho-c-Abl) was then diluted in 3 % FBS and 0.1 % Triton X-100 in PBS. Cells were incubated overnight after being thoroughly washed twice with 3 % FBS in PBS. DAPI (nuclear stain) and Alexa Fluor-688 (red) (Thermo Fisher Scientific. Waltham, MA, USA), a secondary antibody, were added to the cells and incubated for 30 min the next day. The last washing step was three washes using 0.1 % Triton X-100 in 3 % FBS and PBS.

Image Acquisition used a 20x magnification with images being taken with the Zeiss Cell Discoverer 7 Imaging System. The picture capture program was Zen Blue 3.7.

Protein Expression Quantification: Immunofluorescence data were used to quantify the relative amounts of phospho-c-Abl and HIF-1α protein expression.

### Statistical analysis

Data were analyzed using Student's *t*-test. Statistical significance was set at a *p-value < 0.01 and **p-value < 0.001.

## Results

A previous study showed that Crizotinib, an ALK and c-Met inhibitor is able to inhibit both wild-type and T315I-mutated Bcr/Abl [20]. In addition, another study reported that Crizotinib, but not Imatinib, was able to overcome soluble factor-mediated drug resistance in CML cells [21]. This was possible in part because Crizotinib could inhibit JAK2 activity. In the present study, normoxic and hypoxic conditions were used to evaluate the sensitivity of CML cell lines to Abl kinase inhibitors such as Imatinib and Crizotinib.

### Induction of apoptosis in chronic myeloid leukemia cells by imatinib and crizotinib under hypoxic conditions

**Figure 1:**
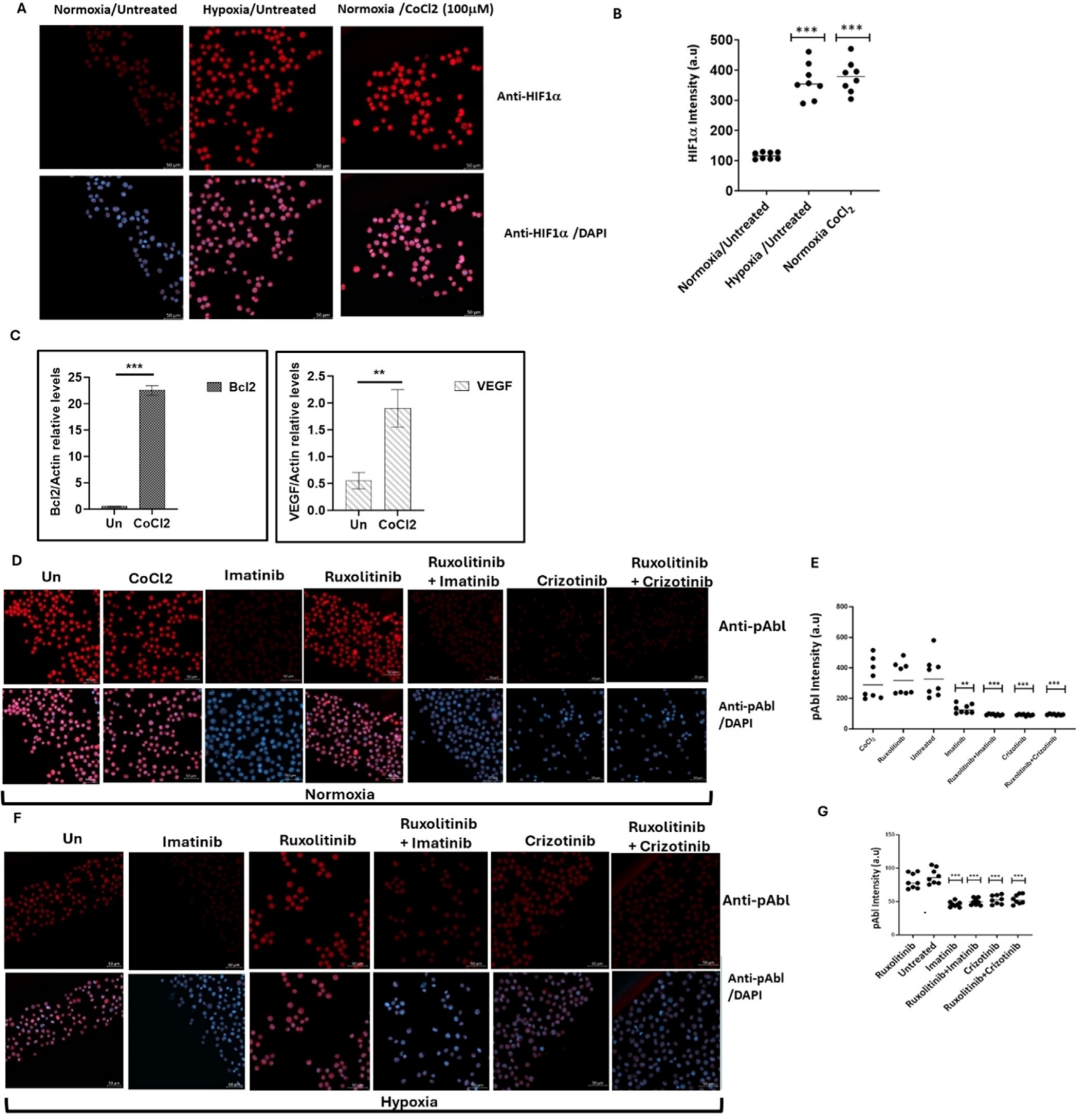
Expression of HIF1α and pAbl in K562 under hypoxic conditions. K562 cells were treated with 100 μM cobalt chloride (CoCl_2_) or grown under low oxygen conditions (2 %) for 12 h. (A) Induction of HIF1α in K562 cells by treatment with CoCl_2_ and exposure to hypoxic conditions for 12 h. (B) Quantitation of HIF1a levels under the different conditions. (C) Real-time PCR was used to determine the relative expression of Bcl2 and VEGF in relation to β-actin in K562 cells exposed to 100 μM CoCl_2_. Sequences of the primers used in this study are shown in Table 1. (D) Immunofluorescence assessment of pAbl levels in K562 cells grown under normoxic, and (E) quantitation. (F) Immunofluorescence assessment of pAbl levels in K562 cells grown under hypoxic conditions after 12 h of treatment, Imatinib, Ruxolitinib, Imatinib/Ruxolitinib, and Crizotinib/Ruxolitinib and (G) quantitation. *p-value ≤0.01, **p-value ≤0.001, *** p-value ≤ 0.0001. The experiment, performed in duplicate, was repeated, with consistent results observed across both independent experiments.

CoCl_2_, a known inducer of HIF1, was used to simulate hypoxic conditions in the K562 and BV173 CML cell lines [22]. Immunofluorescence analysis showed that K562 cells exposed to 100 μM CoCl_2_ exhibited increased levels of HIF1 comparable to exposure to low oxygen (Figure 1A and B). Real-time PCR was also used to monitor the expression of HIF1-responsive genes such as *VEGF* and *Bcl2* (Figure 1C) and to demonstrate the functionality of induced HIF1 in K562 cells treated with CoCl_2_.

Exposure to CoCl_2_ and growth of K562 under hypoxic conditions led to an accumulation of HIF1α (Figure 1A and B), which increased the expression of VEGF and Bcl2 by more than fourfold and twentyfold, respectively (Figure 1C). Figure 1D shows that CoCl_2_ treatment had no effect on Abl phosphorylation in K562. The study also showed that Imatinib and Crizotinib alone or in combination with Ruxolitinib inhibited the phosphorylation of Abl under normoxic (Figure 1D and E) and hypoxic (Figure 1F and G) conditions. Interestingly, pAbl levels were lower in hypoxic conditions (Figure 1E) compared to normoxic conditions (Figure 1G).

**Figure 2:**
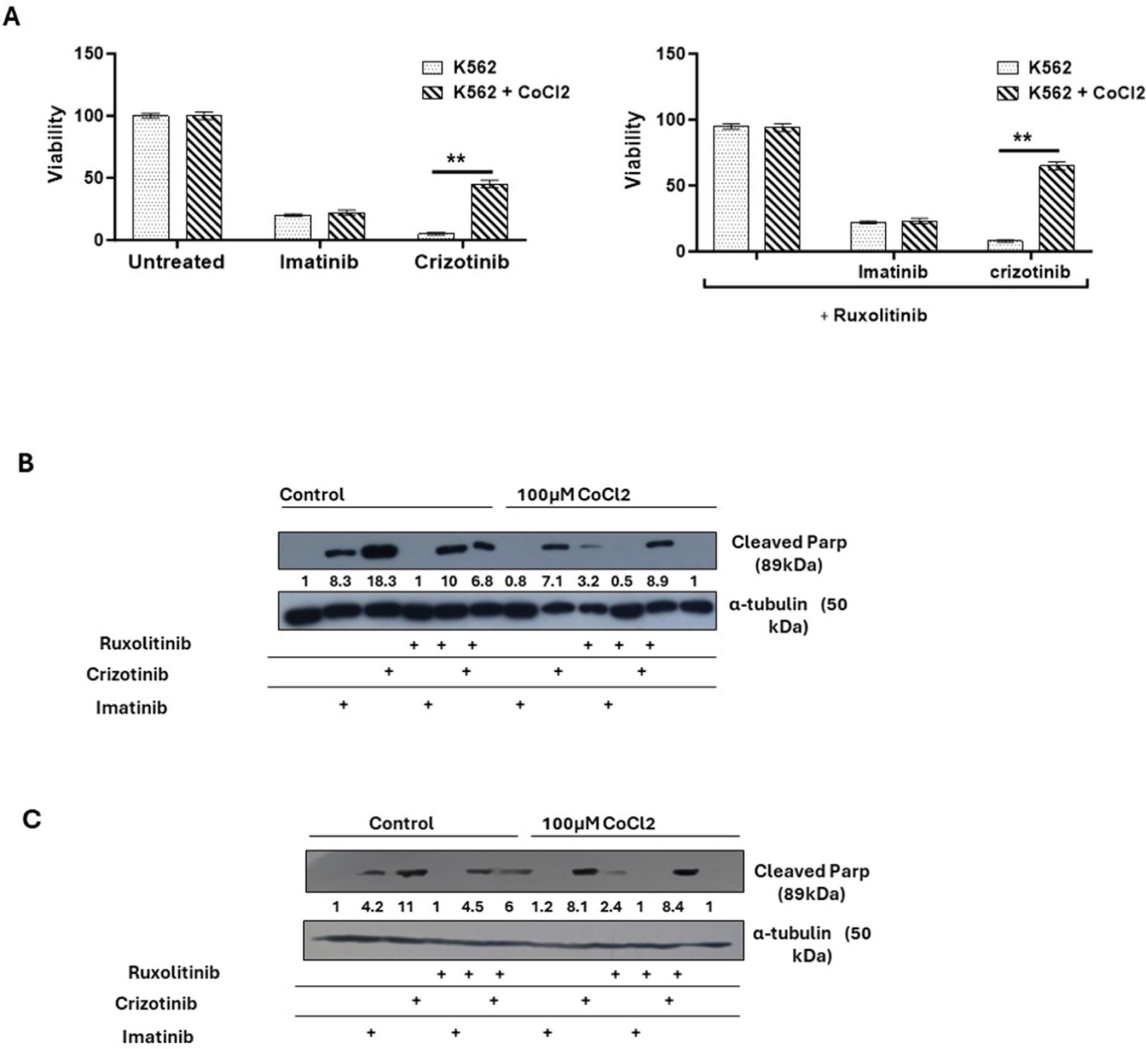
Effect of cobalt chloride (CoCl_2_) and ruxolitinib on the sensitivity of K562 and BV 173 cells to treatment with Imatinib or Crizotinib. K562 or BV 173 cells exposed to 100 µM CoCl_2_ and treated with 1 % DMSO, 1 μM Imatinib, Crizotinib or Ruxolitinib for 12 h. (A) Measurement of the viability of K562 with the trypan blue exclusion assay using a two-sample *t*-test. Immunoblot of (B) K562 and (C) BV 173 cells exposed to 1 µM Imatinib, Crizotinib, and Ruxolitinib in the presence or absence of 100 µM CoCl_2_. Filters were probed with anti-c-PARP and α-tubulin antibodies. The numbers below the plot represent the relative expression levels normalized to α-tubulin.

Next, how Imatinib and Crizotinib affected the proliferation of K562 cells in the presence and absence of CoCl_2_ was examined. Figure 2A shows that exposure to imatinib inhibited the proliferation of K562 in the presence or absence of CoCl_2_. In addition, a significant inhibition of cell proliferation in K562 cells treated with Crizotinib was observed. However, moderate drug resistance was observed in the presence of CoCl_2_ in Crizotinib-treated K562 cells. This suggests that the presence of CoCl_2_ blocks the inhibition of cell proliferation mediated by Crizotinib but not by Imatinib (Figure 2A). Signal Transducer and Activator of Transcription 3 (STAT3) has been associated with hypoxia-induced chemoresistance of ovarian cancer cells [23]. Therefore, the JAK1/2 inhibitor Ruxolitinib [24], which regulates the activity of STAT3, was used to investigate the role of JAK1/2 in the sensitivity of CML cells to Crizotinib. We wondered whether the addition of Ruxolitinib to Crizotinib in the presence of CoCl_2_ could restore sensitivity. Treatment with Ruxolitinib alone had no effect on the proliferation of K562 cells in the presence or absence of CoCl_2_ (Figure 2B). Furthermore, there was no significant difference in the inhibition of K562 cell proliferation between the combinations of Imatinib and Ruxolitinib (Figure 2B). Interestingly, the combination of Ruxolitinib and Crizotinib did not restore the sensitivity of K562 to Crizotinib. In contrast, the inclusion of Ruxolitinib increased Crizotinib resistance in K562 cells (Figure 2B). Comparable data were also obtained using other CML cells such as BV173 (Figure 2C).

### The role of HIF1α and JAK2 in mediating chemoresistance in chronic myeloid leukemia

**Figure 3:**
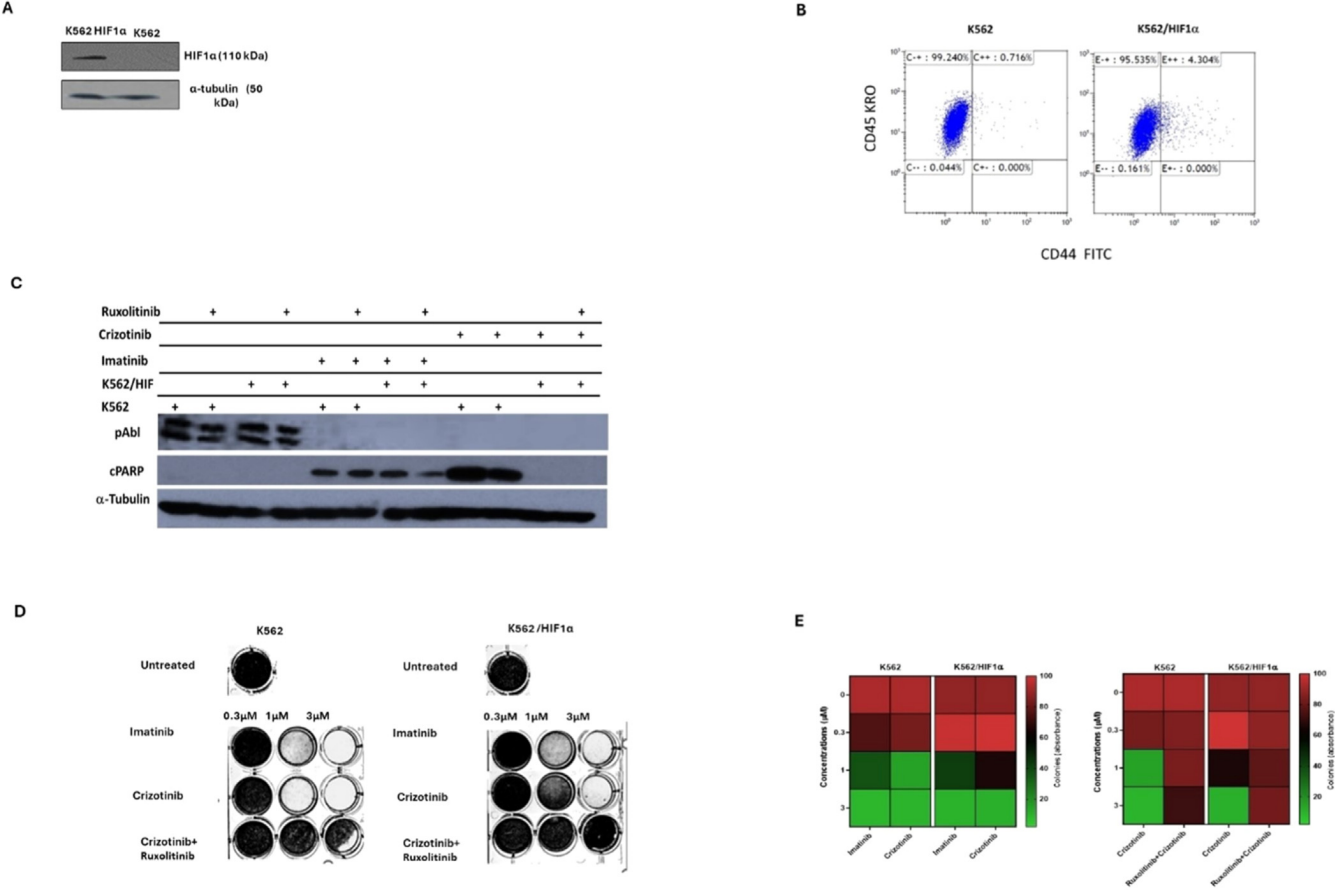
Effect of overexpression of HIF1a on the chemoresistance of K562. (A) Immunoblot of HIF1α in K562 and K562/HIF1α cells. (B) The level of expression of CD44 in K562 and K562/HIF1α was determined by FACS analysis. (C) Levels of pAbl and cleaved PARP in K562 and K562/HIF1α exposed to Imatinib (1μ M), Crizotinib (1μ M), or Ruxolitinib (1 μM) for 24 h. (D) Clonogenicity of K562 and K562/HIF1α cells under semi-solid conditions in the presence of Imatinib (0.3, 1 and 3 µM) and Crizotinib (0.3, 1 and 3 µM) in combination with Ruxolitinib (1 µM). (E) Heatmap of absorptance of dye levels extracted from stained colonies in the different samples. The experiment was performed twice, yielding comparable results.

The above data suggests that the sensitivity of CML cells to Crizotinib is dependent on JAK1/2 activity and hypoxic conditions (Figure 2). However, neither hypoxia nor JAK1/2 activity had a significant effect on sensitivity to Imatinib (Figure 2). HIF1α is an important mediator of hypoxic conditions and modulates the transcription of many genes. To directly demonstrate the role of hypoxia and JAK2 in regulating sensitivity to Crizotinib, a stable mutant of HIF1α (Pro 402 and Pro 564 were replaced by Ala) [25] was overexpressed in K562. Under normoxic conditions, the HIF1a mutant Pro 402 Ala, Pro 564 Ala is not hydroxylated and is therefore not recognized by the von Hippel-Lindau tumor suppressor protein (VHL) for proteasome degradation [26]. Figure 3A shows that HA-HIF1α is overexpressed in K562/HIF1 cells under normoxic conditions. *CD44* is one of the genes regulated by HIF1α [27], and monitoring of levels in K562 and K562/HIF1α cells showed upregulation of CD44 (Figure 3B).

Next, the activity of the Abl inhibitor in K562 cells stably expressing HIF1α was examined. The levels of phosphorylated Bcr/Abl and Abl were essentially unaffected by the overexpression of HIF1α, as shown in Figure 3C. However, the levels of phosphorylated Bcr/Abl and Abl in K562 and K562/HIF1α lysates were completely inhibited by Imatinib or Crizotinib alone or in combination with Ruxolitinib (Figure 3C). This suggests that the ability of Imatinib and Crizotinib to inhibit auto-phosphorylation of the Abl protein is not affected in cells overexpressing HIF1α or exposed to Ruxolitinib. When K562 cells were exposed to Imatinib, PARP was significantly cleaved. Interestingly, the combination of Ruxolitinib and Imatinib decreased the amount of cleaved PARP in K562/HIF1α cells but not in K562 cells. The amount of cleaved PARP was almost twice as high in Crizotinib-treated K562 cells compared to Imatinib-treated cells. In addition, the combination of Ruxolitinib and Crizotinib significantly reduced the amount of cleaved PARP in K562 by about 50 %. In contrast, neither Crizotinib nor Crizotinib in combination with Ruxolitinib resulted in cleavage of PARP in K562/HIF1α cells. Figure 3 shows that HIF1α is responsible for the reduction of cleaved PARP levels in K562 cells when exposed to Crizotinib, although Crizotinib still inhibits Bcr/Abl activity. In addition, it supports the notion that JAK1/2 inhibition contributes to the reduced sensitivity of Imatinib and Crizotinib.

A comparison of the effect of Imatinib and Crizotinib was also evaluated using a clonogenicity assay. Figure 3D shows that Imatinib inhibited colony formation in K562 and K562/HIF1α cells at concentrations of 1 and 3 μM. Crizotinib was more effective in inhibiting colony formation of K562, and significant inhibition was observed at the lowest concentration (0.3 μM). The ability of Crizotinib to inhibit K562/HIF1α was impaired, indicating some degree of resistance. Interestingly, the addition of Ruxolitinib reduced the ability of Crizotinib to inhibit colony formation in both K562/HIF1-positive and -negative cells. Of note, the combination of Ruxolitinib with Crizotinib showed a minimal effect on the proliferation of K562 (Figure 2A - cells grown in two-dimensional [2D] culture) and a significant effect on K562 treated with Crizotinib plus Ruxolitinib in a three-dimensional (3D) culture (Figure 3D).

**Figure 4:**
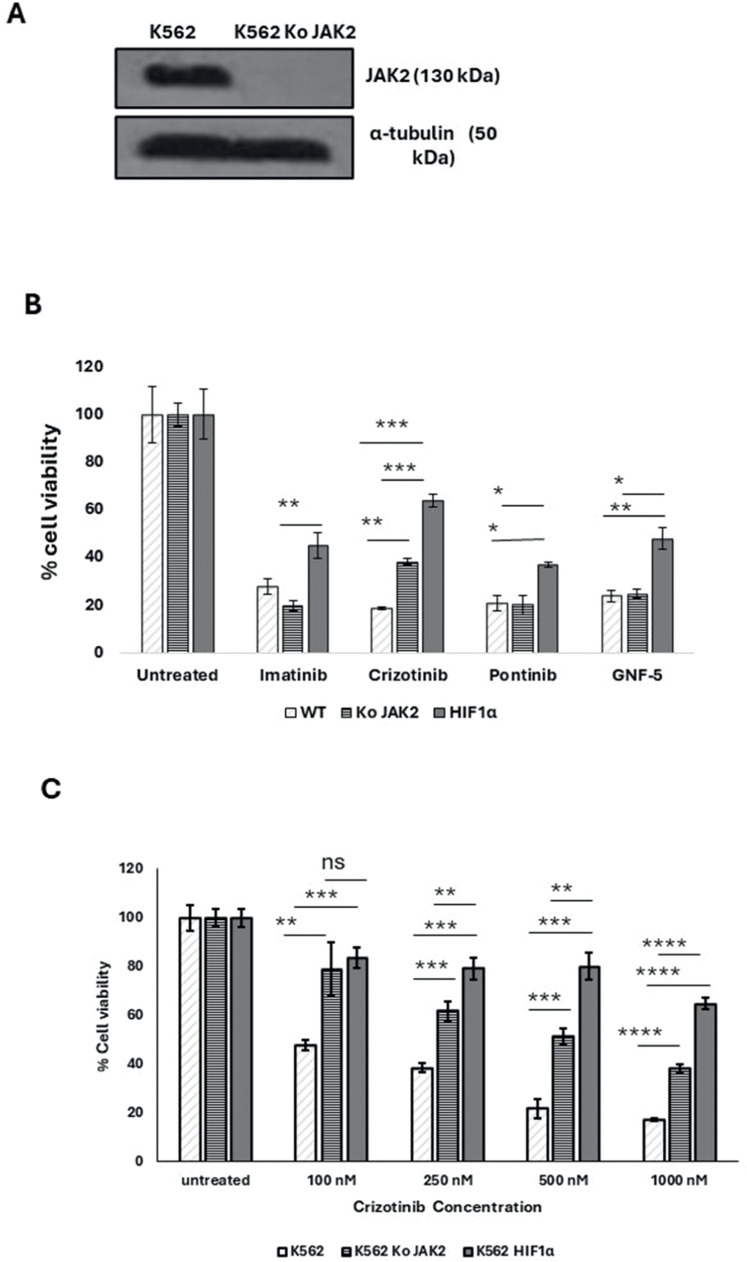
Effect of JAK2 silencing and HIF1α overexpression on the sensitivity of K562 to different Abl kinase inhibitor treatments. (A) Immunoblot of JAK2 in K562 and K562 SiJAK2 cells. (B) Proliferation blot showing remaining K562, K562/Si *JAK2*, and K562 HIF1α cells when exposed to 1000 nM Imatinib, Crizotinib, ponatinib, or GNF-5. (C) Proliferation blot of the different K562 cells that were exposed to different concentrations of Crizotinib. Cell viability was monitored as described in Materials and Methods. *p-value ≤0.01, **p-value ≤0.001, *** p-value ≤ 0.0001. The experiment was run in duplicate and repeated (for a total of two independent experimental runs), with both yielding comparable outcomes.

The *JAK2* gene was also silenced in K562 cells (Figure 4A). The sensitivity of K562, K562/Si *JAK2* and K562/ HA-*HIF*1α P402A/P564A to a range of Abl kinase inhibitors (AKIs) was monitored. The results shown in Figure 4B demonstrate that the different cells have different sensitivities to the different AKIs.

Exposure of various K562 cells to AKIs resulted in a decrease in cell viability. When K562/HIF1α cells were treated with AKIs, a marked and reproducible increase in cell viability was observed compared to K562 cells (Figure 4B), indicating partial drug resistance due to overexpression of HIF1α (Figure 4B). The ability of Crizotinib to inhibit the proliferation of K562 cells was significantly different from that of K562/Si JAK2 and K562/HIF1α cells. Crizotinib was less effective in inhibiting the proliferation of K562/Si JAK2 and K562/HIF1α cells (Figure 4B). Thus, the percentages of viable cells in K562, K562/Si JAK2, and K562/ HA-HIF1α when exposed to 500 nM Crizotinib for 72 h were 23.4, 57.9, and 63.4, respectively (Figure 4C). In addition, no significant differences in cell viability were observed between K562 cells exposed to other AKIs, including ponatinib and GNF-5 (Figure 4B). These results suggest that the reduced sensitivity to Crizotinib is significant in cells overexpressing HIF1α or silenced with JAK2, whereas it is less evident with other AKIs.

### 2-methoxyestradiol restores crizotinib sensitivity of CML cells under hypoxic conditions

The data of this study suggest that HIF1α is responsible for mediating Crizotinib chemoresistance in CML cells. Therefore, it investigated whether HIF1α modulators can restore sensitivity to Crizotinib. Two HIF1α modulators, 2ME2 and Everolimus (Afinitor), were used. 2ME2, a natural estradiol metabolite, has been shown to inhibit the nuclear accumulation and activity of HIF1α in an oxygen- and proteasome-independent manner [28], while Afinitor, an mTOR inhibitor, has been shown to reduce the expression of HIF1α and restore chemosensitivity in ALL cells [29].

**Figure 5:**
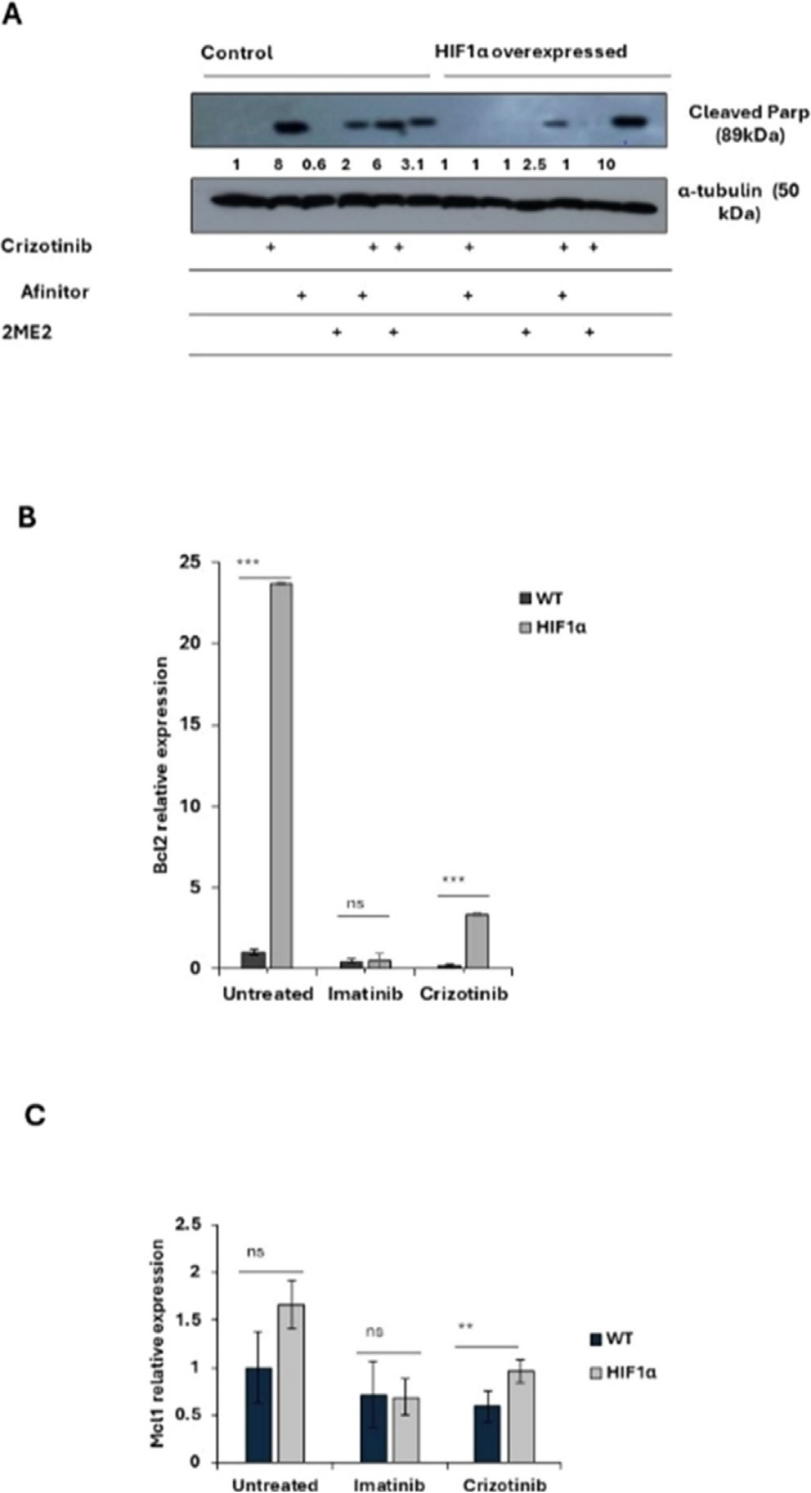
Effect of 2ME2 on HIF1α-mediated resistance to Crizotinib. K562 or K562/HIF1α cells were treated with 1 % DMSO, Imatinib, Crizotinib, Afinitor, or 2ME2 for 24 h. (A) Immunoblot of K562 or K562/HIF1α cells treated with 1 µM Crizotinib, Afinitor, or 2ME2. Filters were probed with anti-c-PARP and α-tubulin antibodies. The numbers below the different lanes represent relative values normalized to α-tubulin. (B and C) Relative expressions of (B) *Bcl2* and (C) *Mcl1* genes compared to the *β-actin* gene were determined by real-time PCR using K562 and K562/HIF1α cells treated with 1 % DMSO, Imatinib, or Crizotinib for 24 h. Sequences of the primers used in this study are shown in Table 1. The levels of PCR amplicons were analyzed using a two-sample *t*-test. The mean relative expression (log2) ± standard error of the mean is shown. *p-value ≤0.01, **p-value ≤ 0.001 and *** p-value ≤ 0.0001. The experiment was performed twice, yielding consistent results.

Figure 5A shows that Crizotinib induces PARP cleavage in K562 cells but not in K562/HIF1α cells. However, 2ME2 was only marginally active in inducing PARP cleavage in K562 cells, whereas Afinitor was not. The combination of Afinitor with Crizotinib had little effect on the sensitivity of K562 cells to Crizotinib, particularly in K562/HIF1α cells. In contrast, the combination of 2ME2 and Crizotinib significantly increased the amount of cleaved PARP in K562/HIF1α cells, suggesting that 2ME2 can restore the sensitivity of Crizotinib in K562 cells overexpressing activated HIF1α. Thus, the decreased sensitivity of CML cells to Crizotinib under hypoxic conditions is primarily mediated by the stabilization and increased activity of HIF1α, and inhibiting the function of HIF1α may restore the sensitivity of Crizotinib in hypoxic CML cells.

To explore the possible mechanism by which activation of HIF1α confers chemoresistance to CML cells despite complete inhibition of Bcr/Abl, changes in apoptosis-related gene expression were monitored in K562 and K562/HIF1α cells treated with Crizotinib. The expression of *Mcl1* and *Bcl2* genes in K562 and k562/HIF1α cells treated with Imatinib or Crizotinib were monitored (Figure 5B). Treatment of K562 cells with Imatinib resulted in a small reduction in the levels of both genes. Treatment with Crizotinib was more effective in reducing the levels of the *MCL1* and *Bcl2* genes. In contrast, treatment with Imatinib in K562/HIF1α led to a similar reduction in the levels of both genes as in K562 cells. However, the levels of *Bcl2* and *MCL1* were significantly higher in Crizotinib-treated K562/HIF1α cells than in Imatinib-treated cells. This suggests that the levels of the two anti-apoptotic genes *Bcl2* and *MCL1* are relatively higher in K562/HIF1α exposed to Crizotinib compared to Imatinib treatment and likely contribute to the chemoresistance to Crizotinib observed in CML cells under hypoxic conditions.

## Discussion

Crizotinib inhibits native and mutant Bcr/Abl kinase activity and shows significant anti-CML activity in vitro and in vivo [30]. In addition, Crizotinib, unlike Imatinib, can effectively overcome the TME-mediated chemoresistance in CML cells, which was attributed to the ability of Crizotinib to inhibit *JAK2* activity [21].

This study investigated the anti-CML activity of Imatinib and Crizotinib under hypoxic conditions. First, it discovered that hypoxic conditions or exposure to CoCl_2_, which mimics hypoxic conditions, impaired the ability of Imatinib to promote apoptosis and inhibit proliferation (Figure 2). Nevertheless, the effect of hypoxic conditions on Crizotinib-treated CML cells was profound and much stronger than that of Imatinib (Figure 2). The effect was not limited to inhibition of cell proliferation of CML, but also included the ability of treated CML cells to undergo apoptosis as measured by monitoring levels of cleaved PARP (Figure 2C and D). The observed effect was not limited to K562 cells, but also other myeloid cells, such as BV173 cells, showed a reduced ability of Crizotinib to induce apoptosis under hypoxic conditions compared to Imatinib-treated BV173 cells (Figure 2). The hypoxia-induced chemoresistance of Crizotinib in CML cells was further enhanced by the combination with Ruxolitinib (JAK1/2 inhibitor) (Figure 2). These results suggest that hypoxia-mediated Crizotinib resistance in CML cells is a phenomenon that is not restricted to a specific CML cell line.

Hypoxia is one of the intrinsic features of solid tumors and is associated with aggressive phenotypes such as resistance to radiotherapy and chemotherapy, metastasis, and a poor patient prognosis [31]. The BM microenvironment is a hypoxic environment that supports hematopoietic stem cells and contributes to leukemia stem cell persistence [12]. In CML, Imatinib inhibited Bcr/Abl activity in hypoxic stem cells, but apoptosis was partially inhibited [32]. Hypoxic conditions have been reported to induce resistance to Crizotinib in NSCLC cell lines with the EML4-ALK rearrangement by activating the EMT process [32]. Hypoxia could induce drug resistance via HIF1-dependent and HIF1-independent mechanisms [33,34]. To corroborate the findings of this study and provide evidence that Crizotinib-induced chemoresistance in CML cells is dependent on HIF1α, the mutant *HIF*1α was overexpressed in K562 cell lines while silencing JAK2. The mutant *HIF*1α (substitution of Pro 402 and Pro 564 by Ala) [25] is not subject to hydroxylation and proteasome degradation [26] and is therefore active under normoxic conditions. K562/HIF1α cells were almost completely resistant to Crizotinib but not to Imatinib (Figure 2, Figure 4), whereas K562 Si JAK2 cells were partially resistant to Crizotinib but not to Imatinib or other AKIs (Figure 4). Remarkably, the data of the current study show that Ruxolitinib, a JAK1/2 inhibitor, enhances the chemoresistance of Crizotinib under hypoxic conditions.

Of note is the ability of Ruxolitinib to enhance the chemoresistance of Crizotinib in K562 cells grown in a 3D culture (Figure 3D), whereas the effect of Ruxolitinib in a 2D culture was minimal (Figure 2A). It is well documented that cells cultured in 3D respond differently to drugs compared to cells cultured in 2D [35]. One explanation could lie in the local pH values within 2D and 3D cells, as lower pH values were found in 3D cells [36]. It is well established that lower intracellular pH values reduce the efficacy of drugs and thus contribute to drug resistance [36]. The results of this study show that the combination of Ruxolitinib with Crizotinib enhanced the chemoresistance of Crizotinib in K562/HIF1α as well as in K562 cultured in 3D In contrast, in a 2D culture, the effect of the combination of Ruxolitinib with Crizotinib was only observed in K562/HIF1α cells. The data are consistent with other observations showing that cancer cell lines growing in 3D culture are more resistant to chemotherapeutic agents than in 2D culture [37].

To elucidate a possible mechanism responsible for resistance to Crizotinib under hypoxic conditions, the levels of the anti-apoptotic genes *MCL-1* and *Bcl2* were measured. Imatinib inhibited the expression of both anti-apoptotic genes *Bcl2* and *MCL1* in both cell types, although the reduction appeared to be greater in K562/HIF1α (Figure 5B). In contrast, Crizotinib significantly reduced the expression of *Bcl2* and *MCL1* in K562 cells, while increasing Bcl2 levels in K562/HIF1α cells (Figure 5B). It is currently uncertain whether the ability of Crizotinib to upregulate *Bcl2* contributes to its ability to promote chemoresistance in K562/HIF1α cells. Hypoxic conditions inhibit the expression of pro-apoptotic genes (*Bax* and *Bim*) and stimulate the expression of anti-apoptotic genes such as *Bcl-2, Mcl-1*, and *XIAP* [38]. An alternative explanation could be related to the ability of hypoxia to induce EMT in cancer cells [39]. Exposure to Crizotinib induces EMT in NSCLC cell lines, leading to chemoresistance [32]. Therefore, one could speculate that Crizotinib treatment in the context of hypoxia may have a stronger ability to induce EMT in K562 cells than Imatinib, leading to increased chemoresistance in treated cells. This speculation still needs to be confirmed experimentally.

The known HIF1α inhibitor 2ME2 was used to provide further evidence for the importance of HIF1α in mediating Crizotinib chemoresistance. Figure 5 shows that 2ME2 can restore sensitivity to Crizotinib in K562/HIF1α cells. This result is consistent with previous findings that 2ME2 can restore the sensitivity of medullary thyroid cancer (MTC) cells [40]. In addition, 2ME2 has been shown to reverse drug resistance in human breast tumor xenografts [41] and multiple myeloma cells [42]. 2ME2 is a natural metabolite of estradiol devoted to estrogenic activity. The antitumor activities of 2ME2 were mediated by its pro-apoptotic activity, microtubule activity and superoxide production [43]. In addition, 2-hydroxyestradiol, a prodrug of 2ME2, was shown to restore the sensitivity of ovarian cancer cells to platinum, which is mediated by the TME [18,44]. These results showed that the combination of 2ME2 and Crizotinib restored the sensitivity of Crizotinib in K562 cells with active HIF1α and JAK1/2 inhibitor treatment (Figure 5). The underlying mechanism by which 2ME2 restores the sensitivity of Crizotinib is not yet clear. The main effect of 2ME2 appears to be due to disruption of the cellular microtubules required for translocation of HIF1α to the nucleus. Thus, 2ME2 may inhibit the accumulation and activity of HIF1α in the nucleus in a manner that is both oxygen- and proteasome-independent [28]. Consistent with previous studies, the data of the current study show that 2ME2 induces apoptosis in K562 cells [18]. Moreover, 2ME2 inhibits the proliferation of prostate cancer cells by modulating the activity of β-catenin [45]. Furthermore, 2ME2 upregulates death receptor 5 (DR5) and induces apoptosis by activating the extrinsic pathway [46]. Recent reports suggest that the antitumor activity of 2ME2 is mediated by its ability to downregulate a number of genes, including the *Bcl2, BCL-XL* and *c-myc* genes [18,47]. Overall, 2ME2 restores the Crizotinib sensitivity of K562 under hypoxic conditions by inhibiting the activity of *HIF*1α [28], which promotes apoptosis possibly mediated by the overexpression of the *Bcl2, Bcl-XL* and *c-myc* genes [18].

The data of the present study showed that Ruxolitinib, a JAK1/2 inhibitor, can attenuate the effect of hypoxia or HIF1α overexpression in mediating Crizotinib resistance in CML cells. Previous studies have shown that JAK1/2 inhibitors such as AG490 promote the accumulation of HIF1α by inhibiting its hydroxylation [48]. Specifically, JAK1/2 is involved in the activation of prolyl hydroxylase domain enzyme activity in HIF1α, which mediates the hydroxylation of HIF1α and its subsequent degradation by the proteasome [48]. Therefore, the JAK1/2 inhibitor Ruxolitinib may inhibit the degradation of HIF1α and contribute to its stabilization [48]. Other studies have shown that inactivation of JAK1 promotes proliferation of endometrial cancer cells by upregulating the HIF1α signaling pathway [49]. The authors suggested that JAK1 may act as a tumor suppressor [49], and that deletion of JAK1 activates the HIF1α pathway [49]. Jeong et al. [50] suggested that JAK1 interacts with HIF-1/2 and reduces the expression of HIF-1/2 protein under hypoxia; therefore, silencing of JAK1 or pharmacologic inhibition of JAK1 kinase activity by Ruxolitinib leading to upregulation of transcription of HIF target genes under hypoxia. Crizotinib may affect the stability of HIF1α in a JAK2-dependent manner, as suggested by the observation that Crizotinib inhibits JAK2 activity [50]. Since Imatinib does not inhibit JAK2, no stabilizing effect is observed in CML cells treated with Imatinib. Furthermore, the activity of Crizotinib was additive to that of Ruxolitinib, which may suggest that different mechanisms are involved in stimulating the stability and function of HIF1α.

In conclusion, this study shows that hypoxic conditions and the presence of Ruxolitinib contribute to a substantial increase in chemoresistance of CML cells to Crizotinib. Although the ability of Crizotinib to inhibit Bcr/Abl activity remained unchanged, its ability to induce apoptosis in CML cells was diminished. It found that K562 cells overexpressing HIF1α exhibited higher levels of the anti-apoptotic genes *Bcl2* and *MCL-1*, suggesting a possible mechanism of action. In addition, this study showed that the inclusion of the HIF1α inhibitor 2ME2 restored the sensitivity of Crizotinib in CML cells. The findings show that HIF1α enhances chemoresistance of Crizotinib independent of Bcr/Abl kinase activity in CML cells. Consequently, targeting HIF1α and the components of this pathway may be critical for overcoming chemoresistance and the complete eradication of CML. In addition, targeting the crosstalk between JAK1/2 and HIF1α has been shown to be a promising strategy for cancer treatment. This intercellular communication could contribute to drug resistance in hypoxic tumor environments. Inhibitors of JAK1/2 or HIF1α or drugs targeting their downstream signaling pathways have great potential for cancer therapy.

## Funding

No external funding for this project.

## Ethics approval and consent to participate

Not applicable.

## Patient consent for publication

Not applicable**.**

## Data availability

The data that support the findings of this study are available from the corresponding author upon reasonable request.

## CRediT authorship contribution statement

**Lena Avinery:** Conceptualization, Investigation, Methodology, Writing – original draft. **Danielle Regev:** Investigation, Methodology, Writing – review & editing. **Hazem Khamaisi:** Conceptualization, Investigation, Methodology. **Jacob Gopas:** Conceptualization, Writing – review & editing, Formal analysis, Resources. **Jamal Mahajna:** Conceptualization, Writing – original draft, Writing – review & editing, Supervision, Formal analysis, Resources.

## Conflicts of interest

The authors declare that they have no known competing financial interests or personal relationships that could have appeared to influence the work reported in this paper.
